# Primary Pancreatic Signet Ring Cell Carcinoma: A Case Report and Review of the Literature

**DOI:** 10.1089/pancan.2020.0013

**Published:** 2021-01-21

**Authors:** Daniel J. Campbell, Emily L. Isch, Geoffrey M. Kozak, Charles J. Yeo

**Affiliations:** Department of Surgery, Thomas Jefferson University, Philadelphia, Pennsylvania, USA.

**Keywords:** adenocarcinoma, adjuvant therapy, epidemiology, pancreatic cancer, pancreatoduodenectomy, signet ring cell carcinoma

## Abstract

**Background:** Primary pancreatic signet ring cell carcinoma (PPSRCC) is a rare (<1%) poorly reported histopathological variant of pancreatic cancer with ill-defined treatment guidelines. Herein, we describe a case of nonmetastatic PPSRCC in a 45-year-old female.

**Presentation:** A 45-year-old female presented with 3 weeks of abdominal pain radiating to her back. Other pertinent positives included a 20-pound (9.1-kilogram) weight loss and jaundice, with a known 30-pack-year smoking history. CT scan revealed a 4.6 × 3.6 cm hypoattenuating mass in the head of the pancreas (HOP) with dilatation of the common bile duct. Total bilirubin at presentation was elevated, and a biliary stent was placed endoscopically. Subsequent endoscopic ultrasonography revealed a periampullary ulcerated mass involving the HOP and second portion of the duodenum, with pathology revealing poorly differentiated adenocarcinoma with mucinous background and focal signet ring cells. A classic pancreatoduodenectomy (Whipple procedure) was performed. Final pathology revealed a poorly differentiated (G3) pT3/pN2/pM0 PPSRCC with 11 of 16 positive specimen lymph nodes. The tumor had evidence of both KRAS and TP53 mutations and expressed an MUC1+/MUC2-/MUC5AC+ immunophenotype. Medical oncology recommended a 6-month course of adjuvant modified-dose FOLFIRINOX therapy.

**Conclusion:** This report highlights the need for further research into the pathogenesis of gastrointestinal signet ring cell carcinoma to identify and study therapeutic targets that can eventually be translated to PPSRCC treatment. Given the paucity of PPSRCC, adjuvant therapy candidates follow the current literature on more common pancreatic cancer subtypes to guide treatment.

## Introduction

Pancreatic cancer is the third leading cause of cancer-related deaths in the United States, with an estimated 57,600 new cases in 2020.^1^ Although highly dependent on its stage at the time of diagnosis, the overall 5-year survival rate for all types of pancreatic cancer is 9%, relatively low compared with other cancers.^1^ Approximately 65% of pancreatic cancers are in the head of the pancreas (HOP), and resectable tumors of this location can be treated with a pancreatoduodenectomy (Whipple) procedure.^2^ At least nine histopathological variants of pancreatic cancer have been identified, of which adenocarcinoma is the most prevalent (85.8%).^3^ Treatment protocols for pancreatic cancer vary by institution and individual case tumor characteristics, but all generally include one or more of the following modalities: (1) resection, (2) radiation, or (3) systemic chemotherapy. Given the relative high proportion of pancreatic cancers that are adenocarcinomas, many subtypes of pancreatic cancer generally draw on literature specifically related to the adenocarcinoma variant when guiding treatments.^4^

Primary pancreatic signet ring cell carcinoma (PPSRCC) is a rare infrequently reported histopathological variant of pancreatic cancer with an estimated incidence of <1%.^5^ The most recent 2019 World Health Organization classification system defines PPSRCC as a subtype of pancreatic ductal adenocarcinoma.^6^ Recent literature suggests that PPSRCC has a lower overall 5-year survival rate than pancreatic adenocarcinoma as a whole (4% vs. 9%) and is more likely to present with distant disease at the time of diagnosis (69.4% vs. 52%).^5^ Owing to its low prevalence and reporting, treatment guidelines specific for PPSRCC do not exist in the literature, and treatment is guided by existing literature on pancreatic adenocarcinoma. Herein, we describe a case of a nonmetastatic PPSRCC in a 45-year-old female treated with classic pancreatoduodenectomy.

## Case Report

**FIG. 1.**
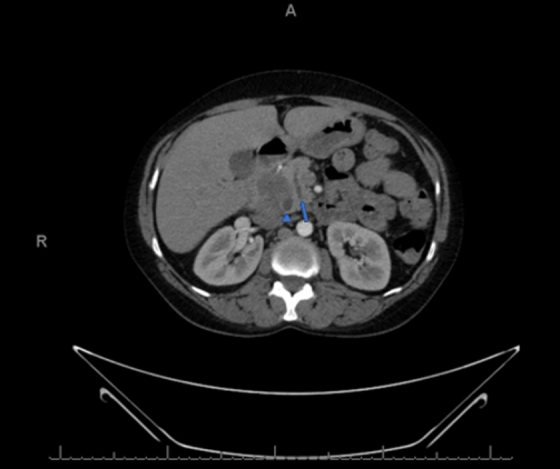
Axial CT image revealing mass in the HOP with CBD (arrowhead) and pancreatic duct (arrow) dilation up to 14 and 3 mm, respectively. CBD, common bile duct; HOP, head of the pancreas.

**FIG. 2.**
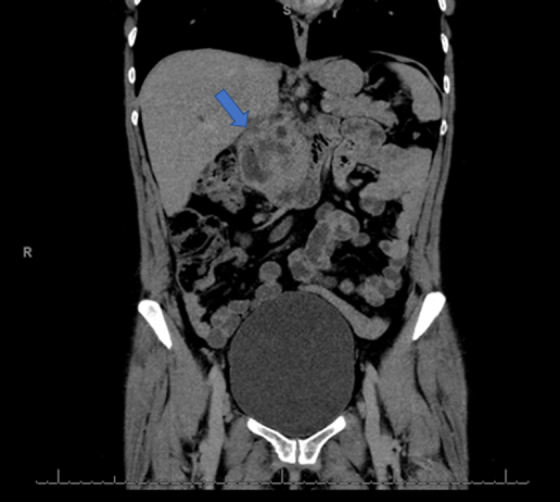
Coronal CT image revealing a 4.6 cm by 3.6 cm hypoattenuating mass (arrow) in the HOP.

A 45-year-old moderately active female presented to our institution for evaluation and management of a periampullary ulcerated mass consistent with poorly differentiated adenocarcinoma with mucinous and signet ring features. The patient was initially seen at an outside hospital 3 weeks before presentation to us, with symptoms of jaundice, weight loss, vomiting, and worsening abdominal pain with radiation to her back. She underwent a CT scan with intravenous contrast that revealed a 4.6 × 3.6 cm hypoattenuating mass in the HOP (Figs. 1 and 2). There was probable obstruction and dilation of the common bile duct (CBD). Minimal distension of the pancreatic duct was seen, measuring up to 3.0 mm. The liver showed no focal mass lesions. She subsequently underwent an esophagogastroduodenoscopy with endoscopic ultrasonography and was found to have a periampullary ulcerated mass with invasion into the HOP. She then underwent endoscopic CBD stent placement. Biopsies taken at that time revealed a poorly differentiated adenocarcinoma with a mucinous background and focal signet ring cells. The primary etiology was unclear. The patient's medical history was notable for anxiety, hypertension, hyperlipidemia, and mitral valve prolapse. Her surgical history was notable for prior cesarean section and tubal ligation.

On presentation to our surgical clinic (through TeleHealth in the setting of the nationwide COVID-19 pandemic), resolving jaundice was suspected. Laboratory studies revealed elevated transaminases, but normal bilirubin levels (total and direct bilirubin of 1.1 and 0.6 mg/dL, respectively). Both carbohydrate antigen (CA) 19-9 and carcinoembryonic antigen (CEA) levels were within the normal range (17 U/mL and 0.9 ng/mL, respectively). The patient was educated regarding her options, which included no operation, bypass alone, surgical resection, chemotherapy, or chemoradiotherapy. Surgical resection was agreed upon and subsequently pursued.

After telehealth-assisted preoperative assessment and negative COVID-19 testing, the patient was explored. The duodenum was adherent to the pancreas and mass, and, thus, we felt it was necessary to sacrifice the pylorus and entire duodenum. After resection, reconstruction consisted of an end-to-side invaginated pancreatojejunostomy, end-to-side hepaticojejunostomy, and a retrocolic-positioned end-to-side gastrojejunostomy.^7^

**FIG. 3.**
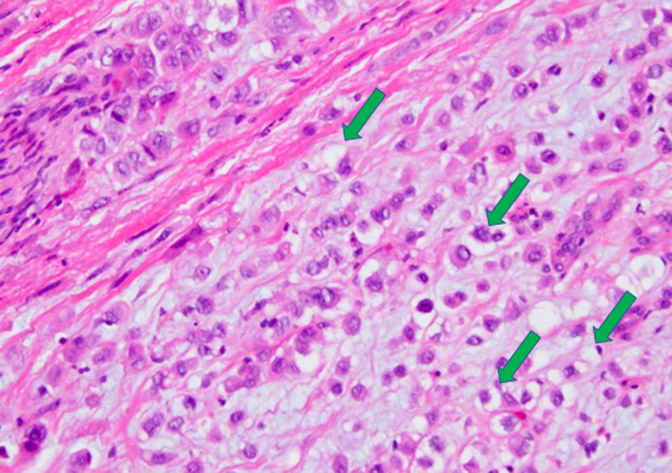
Histology image of resected PPSRCC specimen with characteristic signet ring cells (arrows) comprising >50% of resected specimen. Hematoxylin and eosin stain. 100 × magnification. PPSRCC, primary pancreatic signet ring cell carcinoma.

**FIG. 4.**
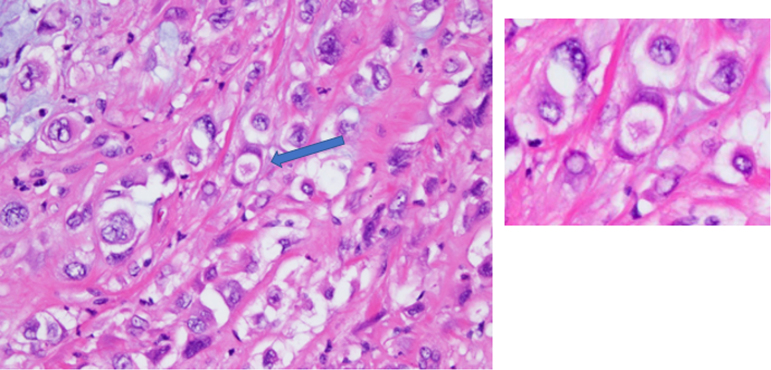
Histology image of resected PPSRCC specimen (left) with emphasis on characteristic signet ring cell (arrow on left, right). Hematoxylin and eosin stain. 400 × magnification.

**FIG. 5.**
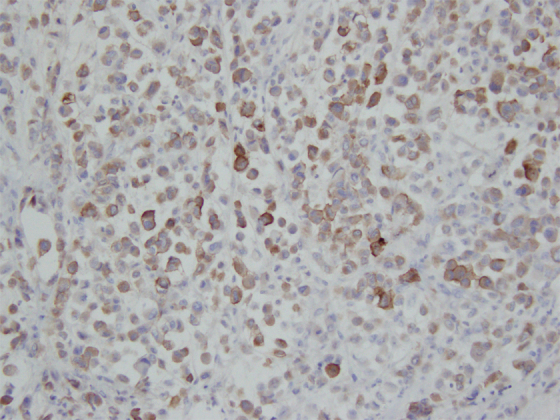
IHC staining of resected PPSRCC specimen demonstrating positive MUC1 expression. 200 × magnification. IHC, immunohistochemical; MUC, mucin.

**FIG. 6.**
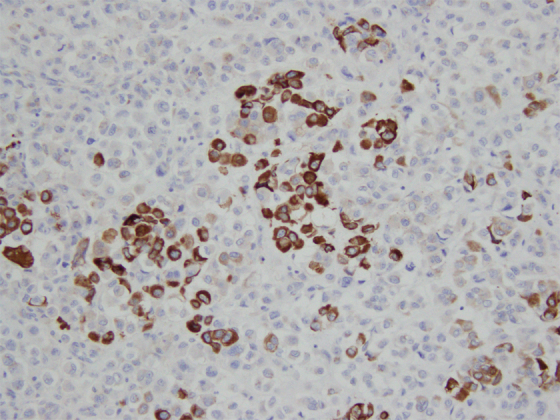
IHC staining of resected PPSRCC specimen demonstrating positive MUC5AC expression. 200 × magnification.

Final pathology for the resected pancreatic mass confirmed poorly differentiated (G3) signet-ring cell carcinoma of probable pancreatic origin (Figs. 3 and 4). The greatest dimension of the tumor was 5.6 cm and did not involve the celiac axis, superior mesenteric artery, or common hepatic artery. There was invasion into the peripancreatic soft tissues and duodenal wall. Eleven of 16 resected specimen lymph nodes were positive for metastatic tumor. All surgical resection margins were reported as negative. The tumor was staged as pT3/pN2/pM0 PPSRCC according to the American Joint Committee on Cancer 8th edition staging system.^8^ The tumor had KRAS and TP53 mutations, and immunohistochemical (IHC) analysis of mismatch repair proteins revealed retained expression of MLH1, PMS2, MSH2, and MSH6. Subsequent IHC staining was performed per protocol to identify mucin (MUC) expression, which revealed positive MUC1 and MUC5AC expression, and negative MUC2, MUC4, and MUC6 expression (Figs. 5 and 6).^9^

The patient recovered uneventfully and per the Whipple accelerated recovery pathway (WARP protocol).^10^ She was discharged home on postoperative day 5 on a full liquid diet. She was quickly advanced to a regular diet and has been progressing well, without any significant complications, as of 2 months postoperatively. She has been seen by a medical oncologist, with plans to initiate adjuvant chemotherapy consisting of modified-dose FOLFIRINOX (mFOLFIRINOX) therapy for an expected duration of 6 months. Germline testing to rule out any DNA damage response deficiencies, such as BRCA and PALB2, was advised and is pending.

## Discussion

PPSRCC of the pancreas is a rarely reported subtype of pancreatic adenocarcinoma with only 10 other case reports found in the literature (Table 1).^11–20^ A recent study analyzing surveillance, epidemiology, and end results program (SEER) data found 497 cases of PPSRCC from 1973 to 2018.^5^ Over 90% of signet ring cell carcinomas (SRCCs) are gastric cancers, and it is estimated that up to 28.2% of primary gastric cancers are in fact SRCCs.^5,21^ Less commonly, SRCCs are found in the breast, gallbladder, bladder, and other locations within the gastrointestinal (GI) tract, including the esophagus, colorectum, and pancreas.^21^ Within the periampullary region, determining the origin of poorly differentiated malignancies can be difficult, and certain MUC staining techniques can aid in determining the origin. MUC staining was performed on our specimen, which revealed expression of MUC1/MUC5AC and lack of expression of MUC2/4/6. Such an expression pattern, particularly the MUC1+/MUC2-/MUC5AC+ immunophenotype, is characteristic of pancreatic adenocarcinoma (Figs. 5 and 6).^22^ Although these findings furthered the supposition that this tissue is pancreatic in origin, it should be noted that this immunophenotype may also be present in cholangiocarcinoma.^22^ Although within the periampullary region, cancers of biliary origin are rarer than that of pancreatic origin, a cancer of biliary origin cannot be completely ruled out.^23^

**Table 1.** Patient Demographics, Laboratory Results, Treatment, and Follow-Up from Existing Case Reports of Primary Pancreatic Signet Ring Cell Carcinoma

First author (year)	Age, gender	Total (direct) bilirubin (mg/dL)	CA 19–9 (U/mL)	CEA (ng/mL)	Treatment	Follow-up
Tracey (1984)	69, M	9.7 (7.0)	n/a	1890	Conservative therapy (SRCC identified postmortem)	Died 13 days after diagnosis
Chow (1994)	88, M	n/a (n/a)	n/a	n/a	Antibiotics (SRCC identified postmortem)	Died 20 days after symptom onset
McArthur (1995)	69, F	0.4 (n/a)	n/a	64	Conservative therapy	Died 7 weeks after admission
Marcy (2002)	39, M	n/a (n/a)	n/a	n/a	Neoadjuvant therapy (3 months), followed by classic Whipple	n/a
Terada (2012)	61, M	n/a (n/a)	WNL	WNL	Resection	No signs of disease progression at 6 months
Karaahmet (2015)	83, M	38.9 (26.8)	15.8	5.5	Not treated (pt refused)	n/a
Nauta (2016)	71, M	37.1 (31.5)	53	n/a	Prednisone (treated for chronic pancreatitis; PPSRCC identified postmortem)	Died (identified postmortem)
Radojkovic (2017)	67, F	30.1 (21.7)	1950	n/a	Three-month neoadjuvant chemotherapy with gemcitabine. Resection	No signs of disease progression at 6 months
Yepuri (2018)	62, F	n/a (n/a)	405	n/a	Not treated (pt refused)	Died 8 weeks after diagnosis
Alexander (2019)	79, F	6.3 (n/a)	334	n/a	Resection	No signs of disease progression at 5 months
Our patient	45, F	0.6 (1.1)	17	0.9	Classic Whipple; 6-month adjuvant mFOLFIRINOX	No signs of disease progression at 2 months

CA, carbohydrate antigen; CEA, carcinoembryonic antigen; mFOLFIRINOX, modified-dose FOLFIRINOX; n/a, not applicable; PPSRCC, primary pancreatic signet ring cell carcinoma; pt, patient; SRCC, signet ring cell carcinoma; WNL, within normal limits.

Pathologically, SRCC is a highly malignant form of adenocarcinoma that produces abundant amounts of MUC, which push the nucleus to the periphery and generate the characteristic phenotype of the “signet ring” cell (Fig. 5). It is hypothesized that these cells originate from constitutive activation of the ErbB2/ErbB3 pathway, leading to unregulated activity of PI3K/MEK1 and downstream disruption of adherens junction proteins, notably E-Cadherin.^24^ This loss of key cell-to-cell interactions has two primary effects in the development of SRCC: (1) formation of a desmoplastic stroma, which facilitates the creation of a tumor growth-enhancing microenvironment, and (2) the generation of the signet ring cell phenotype. It should be noted that the development of a desmoplastic stroma is not unique to SRCC and is in fact characteristic of many carcinomas. However, it is a subsequent mechanism that leads to the generation of the SRCC phenotype, notably constitutive Muc4/ErbB2 interaction. Muc4 is an integral membrane glycoprotein normally located on the basolateral surface of cells that functions to tether membrane-bound MUCs to the cell membrane. Owing to its location on the basolateral membrane, Muc4 is normally physically separated from the ErbB2 tyrosine kinase typically located on the cell's apical surface. Aberrant interaction of Muc4 with ErbB2 allows for unregulated MUC production and the generation of the signet ring cells.^24,25^ SRCC is histologically defined as >50% signet ring cells in the tumor pathological specimen.^26^

The origin of SRCC in the HOP is not well elucidated and several proposed mechanisms exist within the literature. First, given that >90% of SRCCs are of gastric origin, it is suggested that these cancers stem from a heterotopic gastric mucosa nidus in the pancreatic region.^27^ Indeed, there are reports of ectopic gastric mucosa in the periampullary region in SRCC.^28^

Others suggest that these tumors in the pancreas originate from gastric-type metaplasia to the periampullary region that subsequently develops into a neoplastic pancreatic tumor. This theory stems from reports of gastric metaplasia found near the duodenal bulb of peptic ulcer disease (PUD) patients. It is suggested that increased acidity in the duodenal bulb in PUD patients leads to a metaplastic response in which gastric-type epithelium is protective.^26^ The metaplastic response later spreads through the periampullary region to the pancreas, where it acquires neoplastic potential.

A recent study analyzing SEER data from 1973 to 2013 found 497 total cases of PPSRCC, 247 (49.7%) specifically in the HOP.^5^ Compared with other forms of primary pancreatic cancer, this gives PPSRCC a prevalence of <1%. The most common form of pancreatic cancer is pancreatic adenocarcinoma (85.8% of pancreatic cancers); as such, it is useful to draw comparisons between PPSRCC and pancreatic adenocarcinoma.^3^ PPSRCC was shown to have a 5-year overall survival (OS) of ∼4% (compared with 9% in pancreatic adenocarcinoma). PPSRCC was less likely to present as a localized disease than pancreatic adenocarcinoma (3.0% vs. 11%), and more likely to present with distant disease (69.4% vs. 52%). Distant disease at the time of presentation was shown to portend a 62.2% decrease in overall survival when compared with localized/regional tumors. The median ages of presentation for SRCCs appear to drastically vary based on the primary organ, ranging from 40 to 70 years old. In relation to the pancreas, PPSRCC had a median age of presentation of 68 years, slightly younger than the 70-year-old median age of presentation of pancreatic adenocarcinoma.^5^ No research to date has compared levels of blood tumor markers in PPSRCC, such as CA 19-9 and CEA, with other forms of pancreatic cancer. However, existing case reports suggest that reporting is inconsistent and that levels can be highly variable at presentation (Table 1).

Although comprehensive literature regarding treatment of PPSRCC is sparse, SEER data do provide information on whether reported PPSRCCs were treated with pancreatectomy, external beam radiation therapy (EBRT), or both. Localized/regional disease showed improved OS when treated with pancreatectomy or pancreatectomy+EBRT. No effect was noted for EBRT alone. Distant disease showed improved OS with pancreatectomy, pancreatectomy+EBRT, and EBRT alone. Indeed, studies in patients with metastatic disease from the breast, prostate, and kidney have shown that resection and radiotherapy can independently predict improved survival.^29–31^ Unfortunately, SEER data do not currently report the use of concurrent chemotherapy. Given that chemotherapy and radiation therapy are often coadministered in the treatment of pancreatic adenocarcinoma, it is suggested that the isolated improvement in OS of distant disease patients treated with EBRT alone could be at least partially due to contaminant use of chemotherapy.^5,32^ Moreover, it remains difficult to draw conclusions regarding PPSRCC treatment from this analysis given that therapies have considerably improved from 1973, the starting year for this particular cohort. The only stratified temporal analysis done with this cohort combined all localized, regional, and metastatic PPSRCC tumors from 2000 to 2013 and reported that all three treatment modalities (EBRT, pancreatectomy, and pancreatectomy+EBRT) independently improved OS. However, this analysis has significant drawbacks, as localized, regional, and metastatic tumors have considerable differences in clinical outcomes. Unfortunately, this is the only comprehensive analysis of PPSRCC to date.^5^ Given the lack of robust recent data regarding PPSRCC, such a discussion about therapy and survival is only hypothetical.

Surgical resection is currently the only potentially curative treatment for pancreatic cancer.^32^ Unfortunately, it is estimated that only ∼26% of all pancreatic cancers are regarded as curative surgery candidates, and National Cancer Database analyses have shown that only 30% to 40% of stage 1 pancreatic cancer patients undergo surgical resection.^33^ Patients with high risk of recurrence postresection are typically started on an adjuvant chemotherapy regime. Robust data on the use of adjuvant chemotherapy exist for pancreatic adenocarcinoma but are lacking for other histopathological subtypes, including PPSRCC. Table 1 demonstrates this lack of PPSRCC treatment guideline data by displaying the varying treatment strategies implored across the existing case reports. Various adjuvant chemotherapy regimens for pancreatic adenocarcinoma have been investigated, and large-scale studies have identified that combination adjuvant therapies have increased benefits compared with that of monotherapies.^34^ Specifically, in 2018, the Canadian Cancer Trails Group and the Unicancer GI-PRODIGE Group conducted a multicenter international randomized phase III trial comparing gemcitabine monotherapy with FOLFIRINOX as an adjuvant treatment.^35^ The FOLFIRINOX group showed improved median OS (54.4 months vs. 35.0 months). Based on this literature, and the fact that PPSRCC is indeed a subtype of pancreatic adenocarcinoma, our patient was recommended for treatment with adjuvant mFOLFIRINOX for 6 months.

Given the paucity of PPSRCC, it is difficult to expect evidence-based treatment guidelines specifically for PPSRCC in the near future. However, SRCC of the GI tract, specifically gastric SRCC, is much more commonly reported, and future research into the pathogenesis of GI SRCC could offer therapeutic targets that can eventually be studied in PPSRCC.

Beyond resection, there is still no overall consensus for perioperative chemotherapeutic management of gastric SRCC.^36^ Gastric SRCC was classically thought to be less “chemosensitive” than other forms of gastric cancer. However, this literature was largely based on treatment with 5-fluorouricil and platinum-based therapies, and recent literature suggests that gastric SRCC may be uniquely chemosensitive to taxane-based therapy.^36,37^ Given that gastric and pancreatic SRCCs share a common pathogenesis, it can be posited that, similar to gastric SRCC, PPSRCC may be less chemosensitive to the above-mentioned adjuvant chemotherapies studied in pancreatic cancer literature.

Given the complex molecular signaling cascades involved in the development of the signet ring phenotype, current research in chemical inhibitors and small-interfering RNA that target varying parts of this pathology is promising.^38^ Such therapies are experimental at this point and would likely have to demonstrate efficacy in gastric SRCC before demonstrating efficacy in PPSRCC. Further research into targetable parts of the GI, specifically gastric, SRCC pathogenesis is needed to advance the eventual development of PPSRCC therapeutic research.

## Conclusion

PPSRCC is a rare form pancreatic cancer that is more likely to present as distant disease than other pancreatic cancers. Sparse reporting and overall paucity of PPSRCC have resulted in a failure to adequately study this cancer, causing treatment to be guided by literature not specific to PPSRCC. More rigorous research into the pathogenesis of GI SRCCs as a whole is needed into others to develop targeted therapeutic strategies that can eventually be studied in PPSRCC.

## Abbreviations Used

CA: carbohydrate antigen
CBD: common bile duct
CEA: carcinoembryonic antigen
CT: computed tomography
EBRT: external beam radiation therapy
GI: gastrointestinal
HOP: head of the pancreas
IHC: immunohistochemical
mFOLFIRONOX: modified-dose FOLFIRONOX
MUC: mucin
n/a: not applicable
OS: overall survival
PPSRCC: primary pancreatic signet ring cell carcinoma
pt: patient
PUD: peptic ulcer disease
SEER: surveillance, epidemiology, and end results program
SRCC: signet ring cell carcinoma
WNL: within normal limits
